# An analysis framework for clustering algorithm selection with applications to spectroscopy

**DOI:** 10.1371/journal.pone.0266369

**Published:** 2022-03-31

**Authors:** Simon Crase, Suresh N. Thennadil

**Affiliations:** 1 College of Engineering, IT & Environment, Charles Darwin University, Darwin, Northern Territory, Australia; 2 Defence Science and Technology Group, Edinburgh, South Australia, Australia; 3 Energy and Resources Institute, Charles Darwin University, Darwin, Northern Territory, Australia; National University of Sciences and Technology (NUST), PAKISTAN

## Abstract

Cluster analysis is a valuable unsupervised machine learning technique that is applied in a multitude of domains to identify similarities or clusters in unlabelled data. However, its performance is dependent of the characteristics of the data it is being applied to. There is no universally best clustering algorithm, and hence, there are numerous clustering algorithms available with different performance characteristics. This raises the problem of how to select an appropriate clustering algorithm for the given analytical purposes. We present and validate an analysis framework to address this problem. Unlike most current literature which focuses on characterizing the clustering algorithm itself, we present a wider holistic approach, with a focus on the user’s needs, the data’s characteristics and the characteristics of the clusters it may contain. In our analysis framework, we utilize a softer qualitative approach to identify appropriate characteristics for consideration when matching clustering algorithms to the intended application. These are used to generate a small subset of suitable clustering algorithms whose performance are then evaluated utilizing quantitative cluster validity indices. To validate our analysis framework for selecting clustering algorithms, we applied it to four different types of datasets: three datasets of homemade explosives spectroscopy, eight datasets of publicly available spectroscopy data covering food and biomedical applications, a gene expression cancer dataset, and three classic machine learning datasets. Each data type has discernible differences in the composition of the data and the context within which they are used. Our analysis framework, when applied to each of these challenges, recommended differing subsets of clustering algorithms for final quantitative performance evaluation. For each application, the recommended clustering algorithms were confirmed to contain the top performing algorithms through quantitative performance indices.

## 1. Introduction

Cluster analysis is an unsupervised machine learning technique aimed at generating knowledge from unlabelled data [1]. This is achieved through grouping data points in a multidimensional space based on a similarity metric. The desired result is that data points in a grouping or cluster have a natural relationship to one another and a dissimilarity to data points in other clusters.

There are many aspects of cluster analysis that are challenging for practitioners. Clustering is very much a human construct; hence, mathematical definitions are challenging. Even the definition of good clustering is subjective [2]. Numerous clustering algorithms have been proposed in literature with new clustering algorithms continuing to appear. Despite this ongoing effort from the research community, there is no clear best clustering algorithm. Kleinberg [3] goes as far as proposing clustering as a “basic impossibility theorem” through demonstrating no clustering function can satisfy all of a set of three simple properties (scale-variance, richness, and consistency). Thus, trade-offs are inherent in the clustering problem. To make these trade-offs in selecting appropriate clustering algorithms, there needs to be understanding and consideration of the intended application for the analysis. In our previous works, which surveyed cluster analysis in spectroscopy, we found little consistency, evaluation or even justification in the selection of clustering algorithms [4]. This highlighted the need for better practices.

In reviewing literature aimed at assisting in selecting appropriate clustering algorithms, we have observed that there is a significant focus on the characteristics of the clustering algorithms, but limited consideration is given to the user’s goals of the cluster analysis and the characteristics of the target dataset and the clusters it may contain. While it may be assumed or implied that a reader may consider these aspects themselves, this presents a potential point of failure if this is not done with sufficient rigor.

### 1.1 Related works

Despite cluster analysis being such a widely used technique, the efforts in developing an approach or framework for selecting appropriate clustering algorithms have been somewhat limited. As observed in related works, the majority of the focus has been on characterizing and classifying the algorithms themselves and demonstrating their performance for a given application.

Early work by Dubes and Jain [5] in “Clustering techniques: the user’s dilemma” present a foundation to consideration of user’s needs, highlighting that “looking for an *optimum* clustering algorithm is contrary to the nature of the problem” as it ignores the application and implementation aspects. They present several aspects or characteristics for consideration including user options (algorithm parameters), computational cost, and the type of output. They also highlight the shortcomings of comparing algorithms against a single performance criteria as: 1) it cannot capture all the information that can be gleaned from clustering (e.g. the value of a hierarchical output), 2) comparisons are typically made on well-behaved curated datasets, which may not show an algorithm’s abilities on application data, and 3) it ignores practical implementation aspect such as parameter selection, run time, and storage requirements. They also highlight that many new algorithms are presented and evaluated against data and applications where that algorithm performs best. Even with a similar mindset to our thinking, Jain and Dubes’ focus is still on characterizing the clustering algorithms and how to compare them. The gap still exists in how to capture the user’s needs against which the algorithms can be compared.

Despite that early focus on user’s needs, subsequent work has primarily focused on characterization, classification, and comparison of the algorithms themselves. This algorithm centric focus largely highlights the nature of the research domain and its outputs through publications. Many criteria or taxonomies for classifying clustering algorithms have been developed and refined, often starting from Fisher and Ness’s “Admissible Clustering Procedures” [6]. Ackerman, Ben-David and Loker have made significant efforts towards mathematical algorithm characterization and classification with rigorous but narrow contributions focusing on linkage based clustering algorithms [7–9] and randomized clustering algorithms [7]. This classification of algorithms is important as it has been shown that clustering algorithms that follow the same clustering strategy or mechanism tend to result in similar clustering, despite minor variations in the parameters or the objective functions involved [10]. This can assist with reducing the number of individual clustering algorithms to be evaluated.

In addition to these works capturing the characteristics of clustering algorithms and classifying them, there is a suite of studies comparing and evaluating algorithms against real world and synthetic datasets [11–18]. Aldenderfer and Blashfield [19] highlight that the results of these are difficult to compare because each study has evaluated different combinations of data structures and algorithms. They did however identify four data characteristics that appeared to influence performance of the algorithms: the elements of the cluster structure (cluster shape, size, size difference between cluster, and the number of clusters), the presence of outliers, the degree of cluster overlap, and the choice of similarity measure. This highlights the importance of the *characteristic of the clusters* in the data. Recently, Rodriguez et al. [20] conducted a comprehensive study comparing a variety of clustering algorithms against synthetics datasets with varying parameters to understand how algorithms are affected by those characteristics. Despite being comprehensive and valuable, they used equal number of objects per cluster (balanced clusters), and maximum number of features was 200. As we will show later, these fall outside the characteristics of most of the datasets we will evaluate. This demonstrates the challenge in generating a universally applicable comparison. Hence, there is a requirement to consider the specific user’s need and to conduct comparisons for their applications. Our proposed methodology assists in achieving this.

### 1.2 Contribution

Our primary contribution is an analysis framework to evaluate the clustering algorithms against the purpose of the analysis (the user’s needs) and select appropriate clustering algorithms for that application. This differs from existing literature which focuses on characterizing and classifying the clustering algorithms, or produce purely quantitative performance-based comparisons. In our paper, we present a wider holistic approach to the challenge of selecting a clustering algorithm, with a focus on the user’s needs, the data’s characteristics and the characteristics of the clusters it may contain. Our analysis framework utilizes a softer qualitative approach in an analysis framework, drawing on soft systems thinking to identify appropriate characteristics for consideration when matching clustering algorithms to the intended application. Jain [5, 21, 22] has repeatedly emphasized the importance of incorporating domain knowledge into the selection process. We present a practical way to achieve this and add rigor to this selection process.

In addition to the analysis framework itself, we validate our analysis framework through application to four different types of datasets (fifteen datasets in total) with differing analytical purposes. Through this, we identify a collection of selection criteria relevant for those applications and conclude with recommending the clustering algorithms best suited for the cluster analysis of homemade explosives spectroscopy datasets, public spectroscopy datasets, gene expression datasets and the classic machine learning datasets. Our primary focus for this research is for the application to spectroscopy (which form the majority of the validation datasets). However, our successful evaluation and validation of our analysis framework on the gene expression dataset and the classic machine learning datasets indicate it may have wider applicability beyond spectroscopy.

## 2. An analysis framework for clustering algorithm selection

Quantitatively evaluating a large variety of clustering algorithms can be arduous, and as we have shown, quantitative evaluation alone ignores many of the aspects that influence how well an algorithm meets the user’s need for cluster analysis. Our proposed analysis framework aims to narrow down a potentially large and overwhelming set of candidate clustering algorithms to a small subset of algorithms with characteristics that align with the user’s needs. These are then evaluated using quantitative cluster validation metrics to ensure the final selected algorithm achieves good clustering performance outcomes.

**Fig 1:**
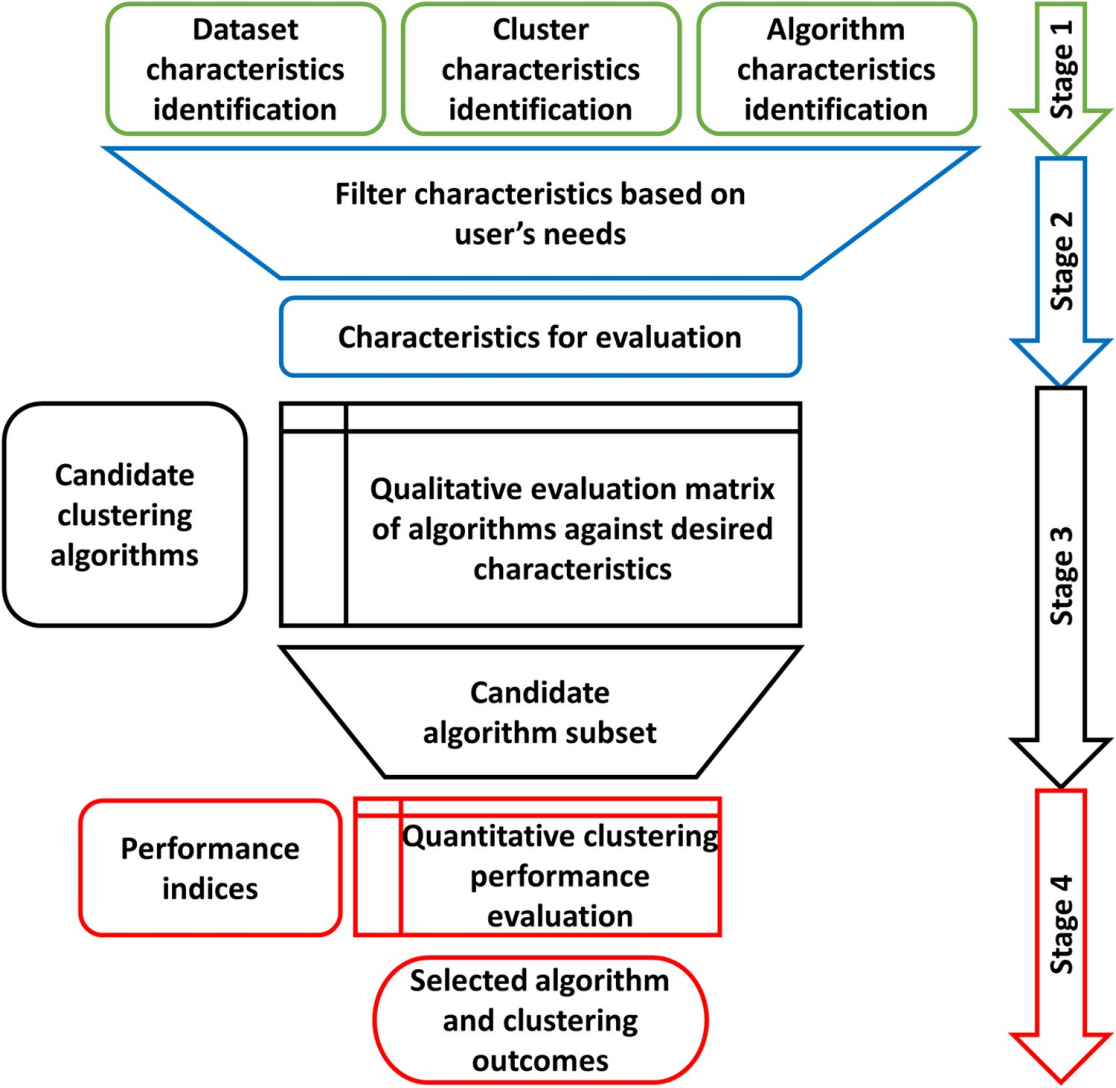
Analysis framework for clustering algorithm selection.

The general workflow consists of generating a set of potentially useful characteristics for consideration (Stage 1). These are then filtered based on the intended application of the cluster analysis (Stage 2). This could be considered the user’s needs and includes the specific characteristics of the data being analysed. These filtered characteristics can then be used to evaluate the range of candidate clustering algorithms and select a subset of algorithms that is likely to satisfy the purpose of the cluster analysis and the user’s needs (Stage 3). Finally, these algorithms are applied to the intended dataset and a final clustering algorithm is selected based on quantitative performance measured against cluster validation indices (Stage 4). This process is summarized in Fig 1 and the detailed steps of each stage are described as follows.

### 2.1 Stage 1—Evaluation characteristics identification

The first step in our analysis framework for selecting cluster algorithms is to identify important characteristics to assess how well a clustering algorithm is likely to meet the needs of the analysis. They may relate to the expected performance outcomes (how well the algorithm can correctly cluster the data), or practical considerations such as the ease of implementation or if the output format of the algorithm meets the purpose of the clustering analysis.

While previous work in developing cluster analysis classification criteria has focused on mathematically definable criteria such as *outer or inner consistent*, *order invariant*, *and k-rich* [7], these appear at odds with the common language terms that are used to describe the characteristics of the datasets and clustering algorithms. We have identified three sources of these evaluation characteristics: the characteristics of the subject data, the characteristics of the clusters contained within the data, and the characteristics of the clustering algorithms. While it may be tempting to assume a generic or universal list of characteristics could be used, we believe these would miss characteristics specific to the user’s application. Hence, generation of these characteristics is an important task in delivering results tailored to the intended analysis application. Our three proposed sources of characteristics are described as follows.

#### 2.1.1 Dataset characteristics

Dataset characteristics refers to the characteristic of the subject datasets (without specific consideration for the clusters it contains). These characteristics are largely dependent on the source of the data. There are numerous frameworks available for data characterization [23] with potential characteristics including the size of the dataset, the number of dimensions (or features), the nature of the data (such as whether it is continuous, nominal, purely numerical, uniformly scaled, or contains missing values), and the presence of labels.

#### 2.1.2 Cluster characteristics

Cluster characteristics refers to the characteristics of the clusters present in the dataset (the cluster structure [19]). Understanding these characteristics typically requires access to labelled data to observe the clusters. This may not always be available, as the typical application of cluster analysis is to cluster unlabelled data. However, there are practical approaches to achieve this if labels are unavailable. These include assessing alternative datasets of a similar type where labels are available or by applying common clustering algorithms to the data itself. Characteristics can also be inferred from the intent of the cluster analysis (e.g., for identification of cancer cells, it can be assumed that there will only be two clusters of interest: cancerous and non-cancerous).

To identify these cluster characteristics, it is helpful to visualize the data and the clusters. We utilize principal component analysis (PCA), an unsupervised dimension reduction technique to reduce the data into its principal components and to visualize the data in two dimensions. An alternative technique is t-distribution stochastic neighbour embedding (t-SNE) [24]. From this two-dimensional visualization of the labelled data, potential observable characteristics include the number of clusters, whether the clusters are non-spherical, varying in density, if there is the presence of noise or outliers, and if the number of points in each cluster is equal (balanced).

#### 2.1.3 Clustering algorithm characteristics

The clustering algorithms characteristics are what are typically discussed when clustering algorithms are compared. These may include characteristics we have already covered under *dataset characteristics* and *cluster characteristics*. However, addressing those separately does sometimes elicit characteristics not often discussed when considering the general characteristics of clustering algorithms.

Beyond the *dataset characteristics* and *cluster characteristics*, additional *clustering algorithm characteristics* often relate to the way the algorithm works, implementation considerations, and the specifics of its output. The clustering algorithm characteristics can typically be identified through reviewing the literature associated with the candidate clustering algorithms that are being considered for the analysis.

### 2.2 Stage 2—Matching characteristics to user’s needs

A significant focus for our analysis framework, and deviation from other literature, is the explicit inclusion of the user’s needs or application needs of the cluster analysis. While this may be implicit in other studies, it will often garner insufficient attention unless it is made explicit. Having the *evaluation characteristics* that we have previously identified enables rigor to be applied to this process.

The general aim of this stage of the analysis framework is to narrow down the set of evaluation characteristics that we have already identified to a set of those that matter for our specific application. While some of this may have already been done intuitively when selecting the datasets characteristics, cluster characteristics and clustering algorithm characteristics, there are likely some that need further consideration. As an example, *computational complexity* is often highlighted as an algorithm characteristic. However, it is not a relevant consideration for small datasets where computation times are short. To include the *computational complexity* characteristic in the selection criteria for those applications may skew the results away from those best suited to this specific application. Hence, the need to match characteristics to the specific user’s needs.

In terms of the practical task of matching a set of evaluation characteristics to the specific needs of the user, this can be as simple as reviewing all the characteristics that have been generated and, with a strong focus on the intended application of the analysis, eliminating those that are not of significant importance. If more rigor or fidelity is required in the application of our analysis framework, weightings could be generated for each evaluation characteristic through techniques such as the analytical hierarchy process (AHP) [25] which utilizes pairwise comparisons.

### 2.3 Stage 3—Qualitative evaluation of candidate algorithms

Once a set of refined characteristics has been selected based on the needs of the application, these can be used to evaluate a set of candidate clustering algorithms as to how well they suit the intended application. At the simplest level, this can take the form of a comparative matrix where candidate clustering algorithms are compared and assessed against the desired characteristics. A simple yes/no evaluation against each category can then be used to guide the analyst to down-select appropriate algorithms.

If further sophistication is desired, a multi-criteria decision making (MCDM) process could be used where weightings are applied to each criteria based on the application needs of the cluster analysis and scores can be given as to how well each candidate algorithm meets each criteria. However, in practical terms, a simple matrix representation of the capabilities against the selection criteria should enable the analyst to down-select a subset of several candidate clustering algorithms for final quantitative performance evaluation.

### 2.4 Stage 4—Quantitative evaluation of selected algorithms

Once a reduced subset of the candidate clustering algorithms has been selected, they can be evaluated using quantitative clustering performance metrics. The use of these quantitative performance metrics enables the final selection of a high performing clustering algorithm that is well suited to the user’s needs. The results of the quantitative evaluation may show that there are several algorithms that perform well. Then, the user can review the algorithm characteristics to determine if they have a preference between the algorithms. Alternatively, cluster ensemble techniques [26, 27] can be used to merge the results of multiple suitable clustering algorithms.

The output of the above-described analysis framework is identification of a cluster analysis algorithm (or several algorithms) that perform well and have characteristics that are well suited to the intended application of the cluster analysis.

## 3. Materials and methods

To validate our analysis framework for cluster analysis algorithm selection, we apply it to four types of datasets with differing characteristics and an associated scenario to add context to the analysis. The primary focus of the analysis is for application to spectroscopy, but additional types of data have been evaluated to indicate the extensibility of our analysis framework. The intent is that the analysis framework will highlight suitable algorithms that meet the needs of each application.

### 3.1 Datasets and context

A range of datasets have been selected with distinct and differing characteristics and applications. Details are as follows:

#### 3.1.1 Homemade detonators explosives spectroscopy datasets

The explosives samples used in this study are representative samples of the homemade explosive detonators used in improvised explosive devices (IED) in the Middle East. Detonators are a small explosive device used to detonate the larger main explosive charge in an IED. The homemade detonators used in this study consist of three stages of explosives of varying chemistries, from which Fourier transform infrared (FTIR) spectroscopy measurements were taken. Thus presenting three spectroscopy data sets for comparison [28]: the *Output Energetic* dataset, the *Transition Energetic* dataset, and the *First Fire Energetic* dataset. The real-world nature of these datasets present unique characteristics for evaluation and differentiation of clustering algorithms. The context of this analysis is to identify relationships between the explosives samples that can infer relationships between the bombmakers that made them.

#### 3.1.2 Public spectroscopy datasets

The publicly available spectroscopy datasets utilized in our study include mid-infrared (MIR), near-infrared (NIR), and Fourier transform infrared (FTIR) spectroscopy and are described as follows: The *Coffee* datasets [29, 30] from two different species, the *Fruit* dataset [30, 31] from adulterated and non-adulterated strawberry purees, the *Liver* datasets [32] annotated according to the majority presence of collagen, glycogen, lipids, or DNA in the cell, the *Mangos* datasets [33, 34] of four different mango cultivars, the *Marzipan* datasets [35, 36] of nine marzipan types, the *Meats* dataset [30, 37] from chicken, pork and turkey mince, the *Olive Oil* dataset [30, 38] of olive oils from four geographic regions, and the *Wine* (FTIR spectroscopy) dataset [36, 39] of wine from four geographic regions. These represent the typical spectroscopy datasets used in laboratory studies where the context is to demonstrate a spectroscopic testing technique can differentiate different materials or families of materials [4].

#### 3.1.3 Gene expression dataset

The gene expression dataset is part of the RNA-Seq (HiSeq) PANCAN dataset from *the cancer genome atlas pan-cancer analysis project* [40]. It contains a random extraction of gene expressions of patients having different types of tumour [41]. This represents the typical high dimensional datasets from aspects of biomedical research.

#### 3.1.4 Classic machine learning (ML) datasets

The classic machine learning datasets are a group of multivariate datasets commonly used within the machine learning community. The *Wine* (multivariate) dataset is used to recognize the wine class given the features like the amount of alcohol, magnesium, phenol, colour intensity, etc. [42]. The *Iris* dataset contains sepal and petal lengths and widths for three classes of plants [42]. The *Breast Cancer* dataset contains ten features extracted from images of benign or malignant breast mass samples [43]. The context of their use (for our demonstration and validation) is for a data scientist learning classification and cluster analysis techniques.

### 3.2 Clustering algorithms and their implementation

To validate our analysis framework, we have selected a range of candidate algorithms for the analysis framework to choose from. There are multiple taxonomies or structures for classifying clustering algorithms [7, 10, 20, 44]. We have broken the domain into *hierarchical*, *partition based*, *density based*, *graph theoretic and spectral clustering*, and *model-based* clustering algorithms and have ensured we evaluate at least one candidate clustering algorithm from each class. Where possible, we have also deliberately selected algorithms with quite differing characteristics to highlight how these may result in differing analysis outcomes, e.g., the selection of Ward’s method and single linkage variants of agglomerative hierarchical clustering. While Ward’s method favours spherical clusters, the single linkage method has a tendency to chain datapoints, or form long, elongated clusters [21]. Hence, both techniques are evaluated in our study to identify if our analysis framework can exploit these differences.

The fourteen chosen algorithms for consideration (and their implementations) are as follows: Hierarchical (Ward’s) [19, 45], Hierarchical (Single Link) [19], BIRCH (Balanced Iterative Reducing and Clustering) [46, 47], *k*-means [48–50], *k*-means minibatch [49, 51], Partitioning around Medoids (PAM) [52], DBSCAN (Density-based Spatial Clustering of Applications with Noise) [49, 53], OPTICS (Ordering Points to Identify Clustering Structure) [49, 54], Mean Shift [49, 55], Spectral Clustering [49, 56, 57], Affinity Propagation [49, 57, 58], and Gaussian Mixture Model [57, 59] were implemented using the *scikit-learn* Python package (https://scikit-learn.org/stable/modules/clustering.html). Fuzzy C-Means [60, 61] was implemented using the Fuzzy C-Means Python package [62] (https://git.io/fuzzy-c-means). HDBSCAN (Hierarchical DBSCAN) [63, 64] was implemented using the hdbscan Python package [64] (https://github.com/scikit-learn-contrib/hdbscan). The associated references contain information about the function of the algorithms and their associated characteristics which are later used in our evaluation process.

Predicting the number of clusters *k* is a significant challenge for cluster analysis. Potential techniques for predicting *k* include the “elbow” method [65], the gap statistic [65], and peak silhouette score [66]. As this is not the focus of this study, the number of clusters *k* was specified from *a priori* knowledge to achieve comparable results across all algorithms. Algorithms were run using their default parameters, except when the algorithm had to be tuned to produce the same number of clusters as other algorithms The detailed configuration and hyperparameter selections are included in the S1 Table.

### 3.3 Data pre-processing

Before the clustering performance of the algorithms is tested, the raw spectra and multivariate data were pre-processed. We utilized extended multiplicative signal correction (EMSC) [67] for spectral data pre-processing which corrects for additive baseline effects, multiplicative scaling effects, and interference effects. The EMSC was implemented for our analysis using Orange3 data mining toolbox in Python [68]. For the multivariate datasets, the data was scaled and centred.

### 3.4 Quantitative evaluation

For the quantitative evaluation of the subset of clustering algorithms, we utilize an external cluster validity index in the form of the V-measure [69]. This assumes data classification labels are available for this evaluation. If data labels are not available, internal cluster validation indices are used such as the silhouette index [66], the Davies-Bouldin index [70], and the Dunn index [71]. Arbelaitz et al. present an extensive comparative study of cluster validity indices [72].

The V-measure external index uses the harmonic mean between the *homogeneity (h)* and *completeness (c)* of clusters. i.e.


$$V_{\beta }=\frac{(1+\beta )*h*c}{(\beta *h)+c}.$$ (1)

The formulations of *h* and *c* are well described by described by Rosenberg and Hirschberg [69]. We use a *β* value of 1 to place equal importance on homogeneity and completeness. The result is a V-measure (VM) score between 0.0 and 1.0, where 1.0 represents perfectly correct labelling. The V-measure was calculated in this analysis using the *scikit-learn* Python package [73].

## 4. Results

As a means of validating our analysis framework for identifying suitable clustering algorithms, we apply it to four differing kinds of datasets and evaluate whether the recommended clustering algorithms suit the intended analytical application (user’s needs).

### 4.1 Stage 1 –Evaluation characteristics identification

The first stage of our analytical framework identifies a set of characteristics from which sub-sets can be selected for the differing applications. These characteristics are drawn from the datasets, the clusters which they contain and the candidate clustering algorithms.

#### 4.1.1 Observed dataset characteristics

As a starting point for characterization, we consider the general characteristics of the four types of evaluation datasets. For our subject datasets (as summarised in Table 1), the dominant characteristics that were identified as worthy of consideration were the *size of the dataset* (number of samples) and the *dimensionality*.

**Table 1:** Characteristics of the validation datasets.

Domain	Dataset Name	Dataset Type	No. of Samples	No. of Features	No. of Classes
Explosives	Output Energetic	Fourier Transform Infrared Spectroscopy	73	3350	5
Spectroscopy	Transition Energetic	Fourier Transform Infrared Spectroscopy	69	3350	8
	First Fire Energetic	Fourier Transform Infrared Spectroscopy	53	3350	7
General (Public)	Coffee	Mid Infrared Spectroscopy	56	286	2
Spectroscopy	Fruit	Fourier Transform Infrared Spectroscopy	983	234	2
	Liver	Fourier Transform Infrared Spectroscopy	731	234	4
	Mangos	Near Infrared Spectroscopy	186	1157	4
	Marzipan	Fourier Transform Infrared Spectroscopy	32	1557	9
	Meats	Fourier Transform Infrared Spectroscopy	120	448	3
	Olive Oil	Fourier Transform Infrared Spectroscopy	120	570	4
	Wine	Fourier Transform Infrared Spectroscopy	44	842	4
Medical	Gene Expression	RNA-Sequence	801	20531	5
Classic Machine	Iris	Multivariate	150	4	3
Learning Examples	Wine	Multivariate	178	13	3
	Breast Cancer	Multivariate	569	30	2

Dataset size is one of the data’s most fundamental characteristics against which clustering algorithms are often compared. Spectroscopy is typically used in laboratories or in process plants where collecting and processing samples can be an expensive process from the perspective of cost, time, and expertise. Hence, the typical number of samples is small and may require specific consideration for suitable algorithm selection. This was observed in the explosives spectroscopy and public spectroscopy datasets.

High dimensionality is another strong trait of spectroscopic data. A large number of measurements are taken at intervals across a spectrum for each sample. Elsewhere, 50 dimensions is referred to as high dimension data for cluster analysis [74]. However, the number of dimensions (features) for spectroscopy is typically in the hundreds or thousands for each sample. The number of variables or features within the gene expression RNA-sequence dataset is an order of magnitude larger again.

Hence, the characteristics we will carry forward for use in our analysis framework are *small datasets* and *high dimensionality*.

#### 4.1.2 Observed cluster characteristics

**Fig 2:**
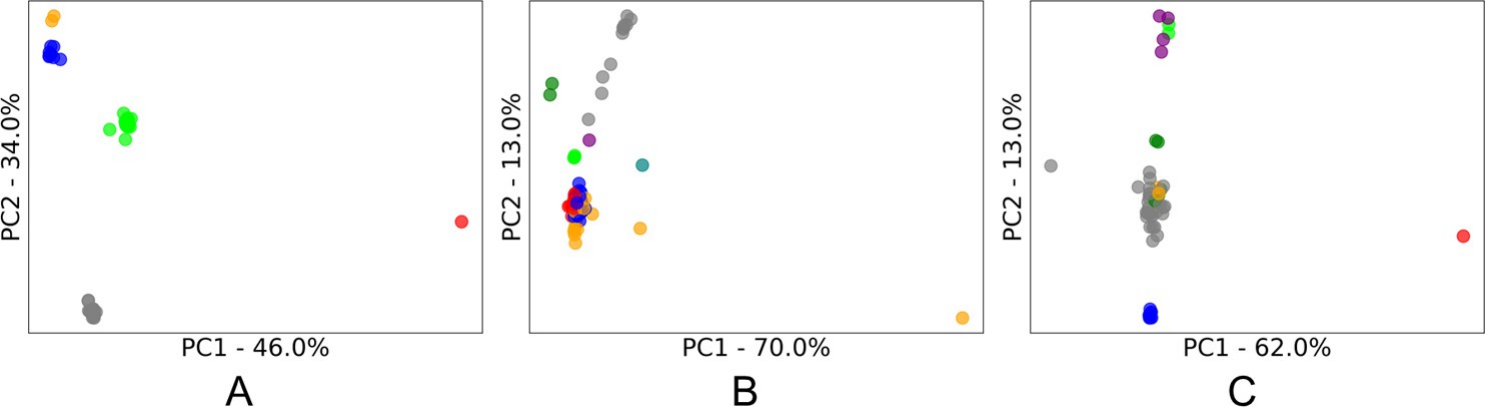
PC1 vs PC2 PCA score plots of the clusters present in the explosives spectroscopy datasets. A: Output Energetic; B: Transition Energetic; C: First Fire Energetic. The percentage of original information contained in each principal component is shown on each axis.

**Fig 3:**
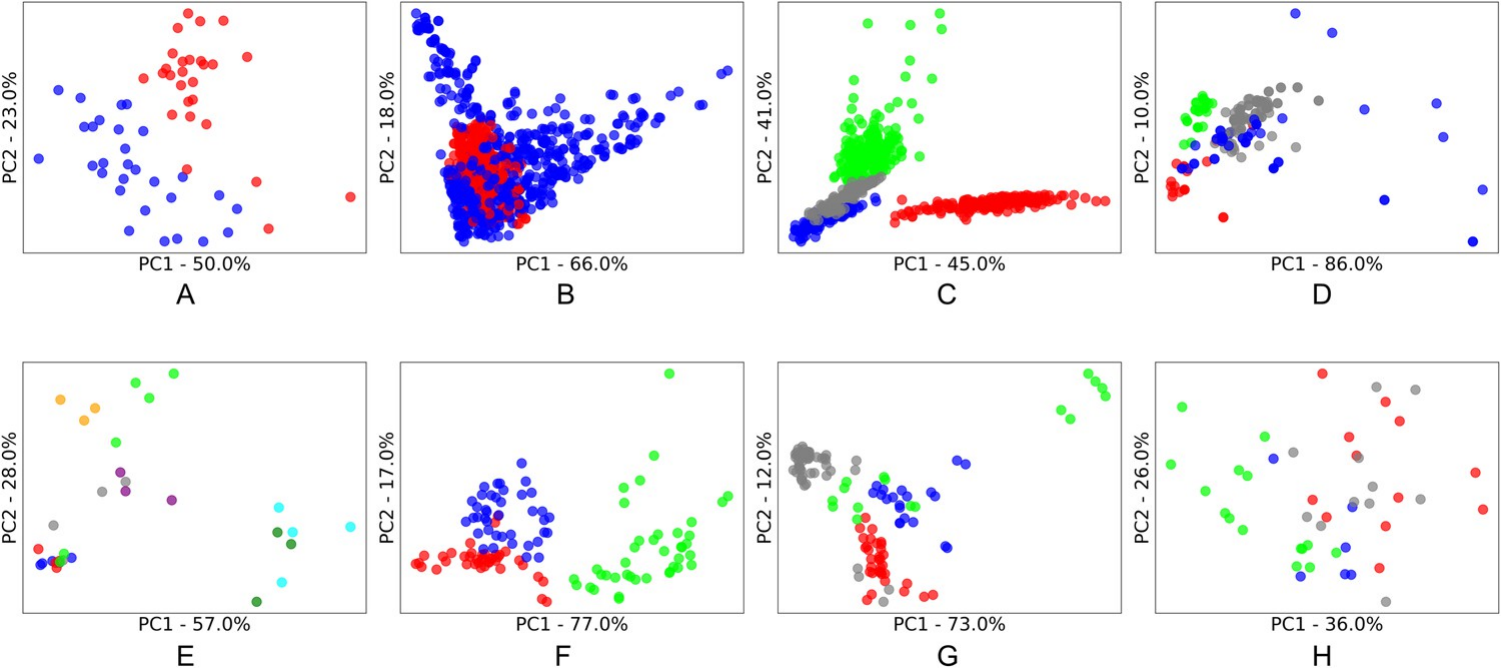
PC1 vs PC2 PCA score plots of the clusters present in the public spectroscopy datasets. A: Coffee; B: Fruit; C: Liver; D: Mangos; E: Marzipan; F: Meats; G: Olive Oil; H: Wine. The percentage of original information contained in each principal component is shown on each axis.

**Fig 4:**
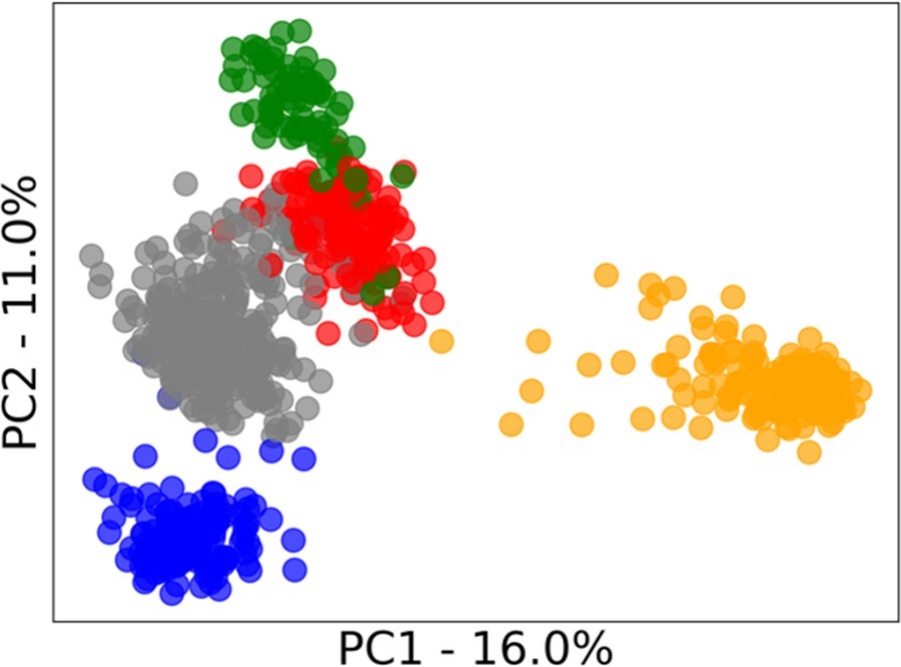
PC1 vs PC2 PCA score plots of the clusters present in the gene expression dataset. The percentage of original information contained in each principal component is shown on each axis.

**Fig 5:**
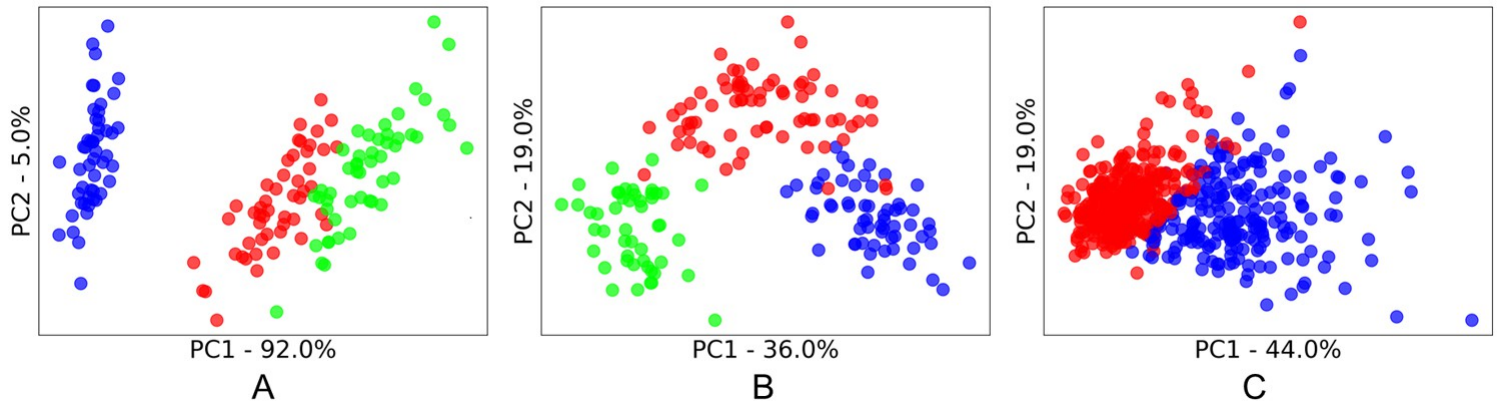
PC1 vs PC2 PCA score plots of the clusters present in the classic ML datasets. A: Iris; B: Wine; C: Breast Cancer. The percentage of original information contained in each principal component is shown on each axis.

As with most aspects of machine learning, an algorithm’s success is often dependent on how well suited it is to the characteristics of specific datasets. For clustering algorithms, this includes the characteristics of the *clusters* contained with the dataset. To understand these cluster characteristics, principal component analysis (PCA) was applied, and the first two principal components were plotted to enable a two-dimensional visualization of the data. True labels of the class of each sample were then applied to see the underlying clusters for each dataset (as shown in Figs 2–5). We acknowledge that these class labels are often not available in applications of cluster analysis (unsupervised learning), and we have included suggestions of alternative methods to infer these characters in section 2.1.2.

The three homemade explosives (energetics) datasets (Fig 2) had noticeably different cluster characteristics to the public spectroscopy datasets (Fig 3) reviewed in our study. These differences likely stemmed from the different purpose of the data samples. As these explosives spectroscopy samples represent real world collections of explosives, they are uneven (unbalanced) in the number of samples of each type of explosive in a dataset. This distribution represents the variation in the explosives encountered in real life, including the possibility of a single sample of one type of explosive being encountered which results in a single sample cluster (or a single point cluster). It was also observed that there is a varying density within certain clusters of homemade explosives, including the possibility of outliers. This may be due to homemade nature of the explosives where ingredients and conditions may vary from batch to batch and result in inconsistencies.

In comparison to the homemade explosives spectroscopy datasets, the public spectroscopy datasets (Fig 3) appear less disparate in the subject matter they are comparing, resulting in less separation between the majority of clusters. The number of datapoints in each cluster appear similar (balanced) with no single point clusters. These characteristics likely stem from the purpose of the testing in the public datasets, i.e., a deliberate chemical testing process for research purposes. The number of clusters or classes within each dataset was relatively small, with Marzipan containing the most at nine clusters. The density of clusters does vary in many of the datasets, both within a cluster and between clusters, and there are some non-spherical clusters. There does not appear to be as many outliers in the public spectroscopy datasets when compared to the homemade explosives dataset, but noise can be observed in the form of scattering or spread in some of the data points.

The gene expression dataset shown in Fig 4 does not present any strong characteristics within the clusters with mostly spherical clusters of equal size (balanced) and minor variations in density and outliers.

The classic ML datasets shown in Fig 5 do not present many strong characteristics within the balanced clusters. The Iris dataset does present elliptical clusters and the breast cancer dataset presents minor variations in density.

In discussing the cluster characteristics of the multiple types of datasets, there were common characteristics that were repeatedly observed, namely *non-spherical clusters*, *varying density within clusters*¸ *unbalanced clusters*, *single point clusters*, *and noise and outliers*. These are the characteristics that we will use in our implementation of our analysis framework for selecting clustering algorithms for the datasets considered here.

#### 4.1.3 Clustering algorithm characteristics

In this section we review the candidate algorithms and capture characteristics for use in our evaluation. Clustering algorithms all generally work to minimize the *within-cluster* distances or maximize the *between-cluster* distances. Each clustering algorithm has a mechanism through which the clusters are generated. These mechanisms impart characteristics on the algorithm as to how well it will work on data with varying characteristics.

In reviewing the candidate clustering algorithms listed in the *Materials and method* section and their characteristics in the associated references, commonly reoccurring characteristics have been identified. These include *high dimensional data*, *non-spherical shaped clusters*, *variable cluster density*, *robustness to outliers and noise*, *multi-modal/hierarchical outputs*, *the number of parameters or hyperparameters*, *deterministic*, and its *time complexity or efficiency*. Many of these have been already identified when considering the dataset characteristics and the cluster characteristics. The additional remaining characteristics that have been captured are now described, including how they can influence algorithm selection.

Multi-modal refers to whether the output of the clustering algorithm produces multiple sets of clusterings (multi-modal) or whether it produces a single set of clustering. Multi-modal algorithms can typically generate a hierarchical output.

The number of parameters or hyperparameters required for implementing an algorithm is of practical consideration. The simplest and most common parameter is the number of clusters (*k*) for an algorithm to generate. Other common parameters for clustering algorithms include the minimum number of points in a cluster (which often influences outlier removal), or hyperparameters relating to minimum densities, distances, or bandwidths. These then influence the resulting number of clusters. When the algorithm is intended for use in an automated process or minimal time is available for implementing hyperparameter tuning, then algorithms with fewest parameters may be preferred due to its ease of implementation.

Deterministic refers to whether the results of each execution of a clustering algorithm are always the same (deterministic) or if a random factor can cause variations in the outcomes and can occasionally deliver poor results. This may require manual intervention or re-running of the algorithm.

Often referred to as time complexity [22], the efficiency of an algorithm affects how long an algorithm takes to execute and the computational and memory requirements. These computational complexities are typically described using the ‘Big O’ notation which captures the order of magnitude in the number of steps to complete the clustering.

Through consideration of the characteristics of the datasets, the clusters that they contain, and the clustering algorithms themselves, we have now developed a set of characteristics for down-selection and use in our evaluation.

### 4.2 Stage 2 –Selection of evaluation characteristics for each application

From the characteristics that we have identified in Stage 1, we will now select those that are most relevant for our specific applications. This is where the differences in the user’s needs and desirable characteristics between each of our four cluster analysis applications will be captured and then, subsequently, used in assessing the candidate clustering algorithms in Stage 3. The characteristics we identified for consideration from the dataset characteristics were *small datasets*, and *high dimensionality*. The characteristics we identified for consideration from the cluster characteristics were *non-spherical clusters*, *varying density within clusters*¸ *uneven cluster sizes*, *single point clusters*, and *noise and outliers*. The additional characteristics we identified for consideration from the clustering algorithms *multi-modal/hierarchical outputs*, *the number of parameters*, *deterministic*, and *efficiency*. We now consider the context of use and expand on the application scenarios to assist in deciding which of these are relevant for each application.

#### 4.2.1 Matching desirable characteristics for homemade explosives spectroscopy analysis

Our analysis of homemade explosive samples utilises spectroscopy data containing real-world influences. These impart unique characteristics onto the dataset and the requirements for the analysis.

From reviewing the general characteristics of the homemade explosives spectroscopy data, small sample size and high dimensionality are clearly relevant.

From reviewing the labelled clusters evident in the homemade explosives spectroscopy datasets, key characteristics were non-spherical clusters (ellipsoidal) of varying cluster density, uneven cluster size, single point clusters, and occasional outliers. Single point clusters are important for this application for when new explosive types are encountered as bombmakers recipes evolve over time. This is somewhat challenging as these points may be considered outliers or noise by some algorithms. Hence, robust to noise and outliers can no longer be a consideration if single point clusters are to be included.

Having a multi-modal (hierarchical) output is especially important for the application of matching homemade explosive samples as the hierarchical output may be used to infer commonalities between bombmakers and potential linkages within a bombmaker network.

A secondary consideration is a desire to create an automated process to regularly reanalyse datasets as more homemade explosive samples are obtained. For this, having deterministic results is attractive as an analysts would not be available to observe intermediate results and manually detect if the random starting points of an algorithm lead to a clear non-optimal local minima and poor results. Additionally, having consistent results between applications would be desirable. Similarly, the use of a minimal number of hyperparameters is desirable if the process is to be automated.

The computational efficiency of an algorithm is less of a concern as these are relatively small datasets, so computation time is low.

From this review, the important characteristics to be used in the evaluation are small datasets, high dimensions, non-spherical clusters, variable cluster density, single point clusters, uneven cluster size, and multi-modal output, with minor consideration given to the number of parameters and deterministic characteristics.

#### 4.2.2 Matching desirable characteristics for public dataset spectroscopy analysis

Cluster analysis of spectroscopy (as seen in the public spectroscopy datasets we evaluate) is often applied in controlled laboratory setting for food, agriculture and biomedical studies [4]. We now consider the characteristics that are important for this application and the associated user’s needs.

From reviewing the general characteristics of the public spectroscopy datasets, small sample size and high dimensionality were clearly evident (Table 1).

From reviewing the labelled clusters evident in the public spectroscopy datasets, key characteristics were non-spherical clusters of varying cluster density, and occasional outliers and noise. Due to the controlled nature of these experimental datasets, they typically contain equal sized clusters with no single point clusters.

Considering the purpose of the analysis and the user’s needs, the multi-modal (hierarchical) cluster analysis capability is important as it enables the results to be related back to the hierarchical nature of the samples (e.g. biological species, genus and family) [4]. However, the number of parameters and the deterministic characteristics of the algorithms are typically not as important as they are unlikely to be performed as part of an automated process. They are more likely to be performed by a researcher or analyst who can tune the parameters, observe the results, and mitigate against poor results due to random starting points for non-deterministic algorithms. Once again, the size of the typical spectroscopy datasets mean computation time is low, hence, computational efficiency is not an important consideration.

From this review, the important characteristics to be used in the evaluation are small datasets, high dimensions, non-spherical clusters, variable cluster density, and multi-modal output.

#### 4.2.3 Matching desirable characteristics for gene expression dataset analysis

Cluster analysis of gene expression datasets presented two core, interrelated characteristics that stem from the nature of the data. The data is extremely high dimensional. This means that algorithms specifically designed for high dimensional data may be attractive. An outcome of this high dimensional data is that the volume of data needing to be processed is large. Hence, efficient algorithms will be attractive to enable analysis within an acceptable computation time without the need for specialist hardware or supercomputer capabilities. The nature of the clusters did not present any strong characteristics as the clusters are balanced, spherical, and of similar density with minimal noise or outliers. The analysis is likely to be performed by a researcher or experienced analyst, so the number of parameters and a deterministic nature are less of a concern. A hierarchical output may be of value depending on the nature of the genomic research being conducted.

From this review, the important characteristics to be used in the evaluation are high dimensions and efficiency.

#### 4.2.4 Matching desirable characteristics for classic ML dataset analysis

The classic ML datasets present an interesting case. For researchers and practitioners to acquire skills in machine learning, datasets are required for learning to work with data, implement code and algorithms, and understand the mathematics and associated phenomena behind it. These classic ML datasets have rose to prominence for this application due to their well-behaved and somewhat simplistic nature and general lack of strong characteristics. I.e., they are of moderate size and low dimensionality which makes them easy to apply and they are typically well curated, so they lack noise, outliers, and contain balanced clusters. They are well-sized, well-balanced, and easily understood datasets.

While the datasets and associated clusters may lack strong characteristics, there are some characteristics that can be implied from how or who is using these datasets. Given that these are datasets are used as a learning and demonstration tool, a primary focus is that they are easy to implement. Hence, a desirable characteristic is that they contain a low number of hyperparameters to tune (preferably only requiring *k*).

The characteristics that we have extracted from the four datasets and their scenarios are summarised in Table 2.

**Table 2:** Characteristics applicable to the validation datasets.

	Dataset Characteristics		Cluster Characteristics				Clustering Algorithm Characteristics				
Dataset Type	Small Datasets	High dimensions	Non-Spherical shape	Variable Cluster Density	Single Point Cluster	Uneven Cluster Size	Robust to Noise and Outliers	Multi-modal (hierarchical)	No. of parameters/ hyperparameters	Deterministic	Efficiency (O)
Explosives Spectroscopy Datasets	X	X	X	X	X	X		X	X	X	
General Spectroscopy Datasets	X	X	X	X			X	X			
Gene Expression Dataset		X									X
Classic ML Datasets									X		

### 4.3 Stage 3 –Qualitative evaluation of candidate clustering algorithms for each application

We now utilise the characteristics we have identified (stage 1) and selected (stage 2) in our analysis framework to evaluate which of the candidate clustering algorithms suits our needs. This is effectively a qualitative process to reduce the large list of candidate clustering algorithms to a select few that are most likely to suit our needs (prior to final evaluation of those selected clustering algorithms based on quantitative clustering performance metrics).

Our approach uses a simple comparative matrix where candidate clustering algorithms are compared and assessed against the desired characteristics. A simple yes/no evaluation against each category can then be used to guide the analyst to down-select appropriate algorithms.

As a starting point for this evaluation, a comparative matrix has been generated for the evaluation characteristics we had previously identified, and the literature associated with the candidate clustering algorithms. This is shown in Table 3. This does not yet take into account the needs of our specific application. *Note that there are some gaps in the information available for certain algorithms against some criteria*. *If this missing information was considered essential in the decision-making process*, *further research could be conducted to assess those algorithms*.

**Table 3:** Comparative matrix of identified characteristics vs. candidate clustering algorithms.

	Dataset Characteristics		Cluster Characteristics					Clustering Algorithm Characteristics			
Clustering Algorithm	Small Datasets	High dimensions	Non-Spherical shape	Variable Cluster Density	Single Point Cluster	Uneven Cluster Size	Robust to Noise and Outliers	Multi-modal (hierarchical)	No. of parameters/ hyperparameters	Deterministic	Efficiency (O)
Hierarchical (Ward’s) [19, 45]	Y	N	N	Y	Y	Y	Y	Y	*k*	Y	*n*^*2*^log *n*
Hierarchical (Single Link) [19]	Y	N	Y	N	Y	Y	N	Y	*k*	Y	*n*^*2*^log *n*
BIRCH [46, 47]	Y	Y	N		Y	Y	Y	Y	*k*+2		*n*
*k*-means [48–50]	Y	N	N	N	Y	N	N	N	*k*	N	*nkdi*
*k*-means minibatch [51] [49]	N	Y	N	N	Y	N	N	N	*k*+1	N	*<nkdi*
PAM [52]	Y	N	N	N	Y	Y	Y	N	*k*	N	*n* ^*2*^ *k* ^*2*^
Fuzzy C-Means [60, 61]	Y	N	N		Y	N	N	N	*k*	N	*nk* ^*2*^ *di*
DBSCAN [49, 53]	Y	N	Y	N	Y^#^	Y	Y	N	2	Y*	*n* log *n*
HDBSCAN [63, 64]	Y	N	Y	Y	Y^#^		Y	Y^+^	2	Y	*n* ^*2*^
OPTICS [49, 54]	Y	N	Y	Y	N	Y	Y	Y^+^	2	Y*	*n*
Mean Shift [49, 55]	Y	N	Y	N	Y	Y	Y	Y^+^	1		*n* ^*2*^
Spectral Clustering [49, 56, 57]	N	Y	Y		Y	N	N	N	*k*+1	N	*n* ^*3*^
Affinity Propagation [49, 57, 58]	Y	Y	Y	N	Y	Y	N	Y^+^	2		*n* ^*2*^ *^i^*
Gaussian Mixture Model [57, 59]	Y	N	Y	Y	Y	Y	Y	N	*k*	N	*nkd* ^*3*^

*n* = the number of objects to be clustered.

*k* = the number of clusters.

*d* = the number of dimensions.

*i* = the number of iterations to convergence.

*Results can change based on the order the data is provided.

+Produces an object such as a minimum spanning tree from which hierarchy can be inferred.

#Enabling single point clustering removes outlier/noise detection capabilities.

This information can now be used for evaluation in matching algorithms to the characteristic needed of each type of dataset and scenario.

#### 4.3.1 Matching desirable characteristics for homemade explosives spectroscopy analysis

The characteristics identified for the evaluation of the homemade explosives (Table 2) are now compared against the candidate clustering algorithms characteristics (Table 3) to enable down-selection of clustering algorithms that best meet the desired characteristics. There was no universal match found against all characteristics, hence, options that meet the majority of characteristic were selected. The clustering techniques down-selected for quantitative performance evaluation on our explosives spectroscopy datasets were hierarchical (Ward’s), hierarchical (single link), HDBSCAN, and affinity propagation.

The hierarchical clustering algorithms appear well suited to this application due to their ability to work with small datasets, single point clusters, uneven cluster size, and produce the hierarchical output that is important for our application. Potential shortcoming could eventuate from the Ward’s methods limited ability to model arbitrary shaped clusters and the Single Link methods limitations on varying density clusters.

HDBSCAN assess well on non-spherical clusters of variable density and can produce a hierarchical output, although it has a limitation of not producing single point clusters and information about its performance on uneven cluster sizes was not readily available.

Affinity propagation clustering is the only selected algorithm explicitly well suited to high dimensional data. However, it is not suited to clusters of varying density and having multiple parameters to tune may make its implementation challenging.

All of the selected algorithms include potential shortcoming for application to the explosives datasets. Hence quantitative clustering performance metrics will be used for final selection of algorithms from this subset (Stage 4).

#### 4.3.2 Matching desirable characteristics for public dataset spectroscopy analysis

The characteristics identified for the evaluation of for the public spectroscopy datasets (Table 2) are now compared against the candidate clustering algorithm characteristics in (Table 3) to enable down-selection of clustering algorithms that best meet the desired characteristics.

In reviewing these characteristics against the clustering algorithm characteristics, the clustering techniques down-selected for quantitative performance evaluation on our explosives spectroscopy datasets were hierarchical (Ward’s), HDBSCAN, OPTICS, and gaussian mixture model clustering.

The hierarchical (Ward’s) clustering algorithm appear well suited due to their ability to work with small datasets, varying density cluster, is robust to outliers, and produces the hierarchical output that is important for our application. HDBSCAN and OPTICS also assess well against these criteria, plus they are well suited to non-spherical shaped clusters and can produce a hierarchical output.

Finally, the gaussian mixture model presents many attractive characteristics. However, it lacks the important ability to create a hierarchical output. Hence, it would require exceptional quantitative clustering performance to offset that limitation. This quantitative clustering performance is evaluated in Stage 4.

#### 4.3.3 Matching desirable characteristics for gene expression dataset analysis

The characteristics identified for the evaluation of for the gene expression dataset (Table 2) are now compared against the candidate clustering algorithm characteristics in (Table 3) to enable down-selection of clustering algorithms that best meet the desired characteristics.

In reviewing these characteristics against the clustering algorithm characteristics, there were four algorithms noted as suitable for high dimension data. However, of those, spectral clustering and affinity propagation were noted for high computational complexity (efficiency (O) above n^2^) and would be unsuitable for our application to the large RNA sequence datasets. Hence, the BIRCH and *k*-means minibatch were selected as they are suited to high dimensional data and very efficient.

#### 4.3.4 Matching desirable characteristics for classic ML dataset analysis

The important characteristics to be used in the evaluation of algorithms for the classic ML datasets was simply minimizing number of parameters (Table 2). This is compared against the candidate clustering algorithm characteristics in (Table 3) to enable down-selection of clustering algorithms that best meet the desired characteristic.

Limiting the selection to the simplest parameter selection (*k*) results in the hierarchical (Ward’s and single link), *k*-means, PAM, fuzzy c-means and gaussian mixture model being selected.

### 4.4 Stage 4 –Quantitative evaluation of clustering algorithms

We have so far highlighted characteristics of clustering algorithms that may make them attractive to the user’s needs and used these characteristics to down-select a subset of clustering algorithms that are well suited to the needs of the analysis. In this section, we quantify the performance of these algorithms on the application datasets. Once the performance of the algorithms is understood, trade-offs can be considered if needed between attractive characteristics and outright performance of the algorithms for our considered application.

In order to validate of our analysis framework, we also perform quantitative evaluation on all of the non-selected candidate clustering algorithms. This will enable comparison between the subset of algorithms that were down-selected as well suited to our application, against those that were not selected. When our analysis framework is being applied in other situations, the intention is to only evaluate the performance of the selected algorithms, hence removing the need for arduous evaluation of a wide range of algorithms.

The clustering algorithms were applied to the four types of datasets (fifteen datasets total) and the results were evaluated using the V-measure external cluster validation index. The results are shown in Table 4, with a total score for each type of dataset presented in bold to enable easier comparisons. The highlighting shows the subset of clustering algorithms down-selected through the qualitative evaluation process (Stage 3).

**Table 4:** V-measure scores for all algorithms and datasets. The highlighted algorithms are those suggested through our analysis framework. The cumulative total score for each type of dataset is shown in the bold columns.

	Explosives Spectroscopy Datasets				Public Spectroscopy Datasets									Gene Dataset	Classic ML Datasets			
Clustering Algorithm	Output Energetic	Transition Energetic	First Fire Energetic	Total Score	Coffee	Fruit	Liver	Mangos	Marzipan	Meats	Olive Oil	Wine	Total Score	Gene Expression	Wine	Iris	Breast Cancer	Total Score
Hierarchical (Ward’s)	1.00	0.71	0.68	**2.39**	0.70	0.08	0.79	0.41	0.81	0.65	0.73	0.37	**4.55**	**0.98**	0.79	0.77	0.46	**2.02**
Hierarchical (Single)	1.00	0.65	0.68	**2.33**	0.03	0.00	0.01	0.10	0.72	0.07	0.22	0.11	**1.28**	**0.01**	0.03	0.72	0.01	**0.76**
BIRCH	1.00	0.71	0.68	**2.39**	0.70	0.08	0.57	0.08	0.81	0.65	0.73	0.37	**3.99**	**0.98**	0.79	0.77	0.46	**2.02**
*k*-means	1.00	0.71	0.73	**2.44**	0.54	0.15	0.75	0.40	0.81	0.81	0.67	0.33	**4.46**	**0.98**	0.89	0.76	0.53	**2.18**
*k* -means minibatch	1.00	0.69	0.74	**2.43**	0.54	0.15	0.75	0.29	0.83	0.67	0.69	0.33	**4.25**	**0.97**	0.86	0.76	0.58	**2.2**
PAM	1.00	0.73	0.73	**2.46**	0.59	0.15	0.73	0.39	0.81	0.58	0.67	0.28	**4.21**	**0.95**	0.78	0.79	0.49	**2.06**
Fuzzy C-Means	1.00	0.63	0.70	**2.33**	0.54	0.12	0.74	0.37	0.84	0.80	0.62	0.33	**4.35**	**0.53**	0.88	0.75	0.56	**2.19**
DBSCAN	1.00	0.65	0.68	**2.33**	082	0.10	0.46	0.54	0.72	0.45	0.56	0.12	**3.68**	**0.75**	0.52	0.66	0.04	**1.22**
HDBSCAN	0.95	0.74^	0.77	**2.47**	0.78	0.18	0.58	0.68	0.84^	0.67	0.49^#^	0.27	**4.49**	**0.73**	0.50	0.73^	0.21	**1.44**
OPTICS	0.36	0.62	0.44	**1.41**	0.69	0.03	0.59	0.56	0.80	0.50	0.53	0.30	**4.00**	**0.77**	0.55	0.63	0.20	**1.38**
Mean Shift	1.00	0.63	0.68	**2.31**	0.06	0.16	0.59	0.20	0.77	0.60	0.50	0.18	**3.06**	**0.04**	0.04	0.77	0.02	**0.83**
Spectral Clustering	0.06*	0.15*	0.13*	**0.34**	0.09	0.12	0.79	0.18	0.86	0.63	0.73	0.29	**3.69**	**0.01** *	0.66	0.77	0.01*	**1.34**
Affinity Propagation	0.64	0.55	0.50	**1.68**	0.57	0.15	0.72	0.07	0.75	0.35	0.62^	0.21	**3.44**	**0.93**	0.56	0.73	0.50	**1.89**
Gaussian Mixture Model	1.00	0.74	0.68	**2.42**	0.65	0.14	0.74	0.36	0.83	0.54	0.67	0.26	**4.20**	**0.82**	0.88	0.90	0.66	**2.44**

^#^large number of noise.

^could not generate specified *k* number of clusters.

* could not generate a complete graph from the similarity matrix, which resulted in incorrect clustering.

### 4.5 Evaluation of the framework for clustering algorithm selection

We now consider the success of the analysis framework in selecting suitable algorithms that are high performing and meet the needs of the analysis for each of the datasets.

#### 4.5.1 Homemade explosives spectroscopy datasets results

The results (highlighted in green in Table 4) for the three explosives spectroscopy datasets show that we had correctly selected multiple high performing algorithms with the Affinity Propagation algorithms performance being the only exception. Hierarchical (Ward’s) produced the best results from the Ward’s and Single link hierarchical clustering algorithms. HDBSCAN produced the best overall clustering performance but is unable to model single point clusters. Thus, depending on priorities, Hierarchical (Ward’s) or HDBSCAN algorithms are recommended for cluster analysis of homemade explosives spectroscopy.

There were other algorithms such as *k*-means, PAM, BIRCH and gaussian mixture model deliver strong clustering results that were not chosen by our analysis framework. These were not selected due to their mismatch to the user’s needs of the cluster analysis. I.e., the *k*-means variants, PAM and gaussian mixture model lack a hierarchical output (which is important for our analytical application), and the higher number of parameters required for the BIRCH algorithm that would make future automated implementations challenging. This highlights the value of our analysis framework for algorithm selection when compared to selection algorithms purely based on quantitative performance-based metrics.

#### 4.5.2 Public spectroscopy datasets results

The results (highlighted in yellow in Table 4) for the eight public spectroscopy datasets show that we had correctly selected multiple high performing algorithms. Hierarchical (Ward’s), HDBSCAN, OPTICS and gaussian mixture model all performed well. Given the highest performance of hierarchical (Ward’s) and the hierarchical output that is well matched to the typical user’s needs when clustering spectroscopy datasets, hierarchical clustering using Ward’s method is our recommended clustering algorithm.

One observation of note is that unlike for the homemade explosives spectroscopy dataset, our analysis framework did not down-select the hierarchical single-linkage method for the public spectroscopy datasets. This choice was vindicated by the poor resulting clustering performance of Hierarchical (Single) algorithm as shown in Table 4. This is likely due to the lack of the elongated chain like clusters that single link hierarchical clustering is best suited to. This indicates correct function of our analysis framework, and that the approach used in our analysis framework is suitable for identifying likely high performing clustering algorithms given a specific user’s scenario.

A second observation of note was that while *k*-means clustering algorithm was the second-best performing algorithm for each set of spectroscopy data, it was not recommended by the analysis framework. This is primarily due to its lack of a hierarchical output, and hence, does not match the typical user’s needs where the public spectroscopy datasets commonly analyse biological materials that contain a taxonomical hierarchy [4].

#### 4.5.3 Gene expression dataset results

The results (highlighted in salmon colour in Table 4) for the gene expression dataset show that we had correctly selected two high performing algorithms for analysis of the gene expression data. BIRCH and *k*-means minibatch both delivered very high v-measure scores and were very fast to compute. The alternative techniques suited to high dimensional data (affinity propagation and spectral clustering) were computationally and memory intensive and hence, were unsuited to this application. Hence, the fact that they were not selected contributes to the validation of our analysis framework.

#### 4.5.4 Classic ML datasets results

Despite having limited guiding characteristics for selecting algorithms for the classic ML datasets, the results (highlighted in blue in Table 4) show that multiple high performing algorithms were selected, including the highest performing gaussian mixture model algorithm. The only selected algorithm to perform poorly was the single link method of hierarchical clustering. This algorithm is best suited to elongated chain like clusters which were not predominant within these datasets.

## 5. Conclusions

We address the challenging task of selecting a clustering algorithm from the many options available. To achieve this, we have presented an analysis framework for selecting appropriate clustering algorithms for specific use cases, where consideration can be made for the intended application of the analysis and the associated desirable characteristics. In contrast to other literature which predominantly focuses on the characteristics of the algorithm, our process considers the characteristics of the data, the characteristics of the clusters it contains, and the characteristics of the clustering algorithm and compares these with the needs of the user (the purpose of the cluster analysis).

To validate our analysis framework for selecting clustering algorithms, we applied it to four different types of datasets with four differing analytical applications. Our analysis framework, when applied to each of these challenges, recommended differing subsets of clustering algorithms for final quantitative performance evaluation. The recommended clustering algorithms were confirmed to contain the top performing algorithms through quantitative performance indices and the algorithms characteristics were well suited the intended context of the analysis (the user’s needs).

For cluster analysis of homemade explosives spectroscopy datasets, we considered the characteristics of small datasets, high dimensions, non-spherical clusters, variable cluster density, single point clusters, uneven cluster size, and multi-modal (hierarchical) output, with minor consideration given to the number of parameters and deterministic characteristics for if the analysis process was to be automated. This resulted in the agglomerative hierarchical clustering (Ward’s method) and HDBSCAN (Hierarchical Density-based Spatial Clustering of Applications with Noise) being assessed as the most suitable clustering algorithms for homemade explosives spectroscopy datasets by delivering high clustering performance while meeting the user’s needs of the cluster analysis.

For cluster analysis of the public spectroscopy datasets, we considered the characteristics of small datasets, high dimensions, non-spherical clusters, variable cluster density, robust to noise and outliers, and multi-modal (hierarchical) output. This resulted in the agglomerative hierarchical clustering (Ward’s method) being assessed as the most suitable clustering algorithms for public spectroscopy datasets by delivering high clustering performance while meeting the user’s needs of the cluster analysis.

For the gene expression dataset, we considered the characteristics of high dimensionality and efficiency in selection of suitable clustering algorithms. Our analysis framework recommended the BIRCH and *k*-means minibatch algorithms which resulted in fast computation on the huge dataset (meeting the user’s needs) and highly accurate clustering outcomes.

For cluster analysis of the classic machine learning datasets, we considered the characteristic of the number of parameters/hyperparameters. This resulted in a selection of multiple high performing algorithms that require the value of *k* (number of clusters) as their only parameter, hence meeting the example user’s needs of simple to implement algorithms.

Cumulatively, these findings indicate that our analysis framework is a valid means of selecting clustering algorithms that are well suited to the user’s needs of cluster analysis. The primary focus in development and evaluation of the analysis framework has been for applications to spectroscopy. The validation of the methodology against eleven varying spectroscopy datasets demonstrates its suitability for this application. However, the successful evaluation of additional types of data (gene expression data and classic machine learning datasets) indicates the analysis framework may be extensible to other clustering applications.

## Supporting information

S1 Table Hyperparameters for each clustering algorithm and dataset. (DOCX)
