# Supplementary material for: An analysis framework for clustering algorithm selection with applications to spectroscopy
Source: PLoS One. 2022 Mar 31;17(3):e0266369. doi: 10.1371/journal.pone.0266369 (PMC8970496; doi:10.1371/journal.pone.0266369)
Supplement: S1 Table — (DOCX) [file pone.0266369.s001.docx]

Supporting information

**S1 Table. Hyperparameters for each Clustering Algorithm and Dataset**

|  | | **Explosives Datasets** | | | | **Public Spectroscopy Datasets** | | | | | | |  | **Gene Dataset** | | | **Classic ML Datasets** | | | |  |
| --- | --- | --- | --- | --- | --- | --- | --- | --- | --- | --- | --- | --- | --- | --- | --- | --- | --- | --- | --- | --- | --- |
| **Algorithm - *hyperparameter*** | | Output Energetic | | Transition Energetic | First Fire Energetic | Coffee | | Fruit | Liver | Mangos | Marzipan | Meats | Olive Oil | Wine | | Gene Expression | Wine | | Iris | Breast Cancer |  |
| Hierarchical (Ward’s) - *k* | | | 5 | | 8 | 7 | 2 | | 2 | 4 | 4 | 9 | 3 | 4 | 4 | | 5 | 3 | | 3 | 2 |
| Hierarchical (Single) - *k* | | | 5 | | 8 | 7 | 2 | | 2 | 4 | 4 | 9 | 3 | 4 | 4 | | 5 | 3 | | 3 | 2 |
| BIRCH - *k* | | | 5 | | 8 | 7 | 2 | | 2 | 4 | 4 | 9 | 3 | 4 | 4 | | 5 | 3 | | 3 | 2 |
| BIRCH – *branching_factor* | | | 50 | | 50 | 50 | 50 | | 50 | 50 | 50 | 50 | 50 | 50 | 50 | | 50 | 50 | | 50 | 50 |
| BIRCH – *threshold* | | | 0.5 | | 0.00049 | 0.005 | 0.5 | | 0.0005 | 0.5 | 0.5 | 0.0005 | 0.01 | 0.0005 | 0.0005 | | 0.5 | 0.0005 | | 0.0005 | 0.0005 |
| *k*-means - *k* | | | 5 | | 8 | 7 | 2 | | 2 | 4 | 4 | 9 | 3 | 4 | 4 | | 5 | 3 | | 3 | 2 |
| *k* -means minibatch - *k* | | | 5 | | 8 | 7 | 2 | | 2 | 4 | 4 | 9 | 3 | 4 | 4 | | 5 | 3 | | 3 | 2 |
| *k* -means minibatch – *batch-size* | | | 200 | | 200 | 200 | 200 | | 200 | 200 | 200 | 200 | 200 | 200 | 200 | | 200 | 200 | | 200 | 200 |
| PAM - *k* | | | 5 | | 8 | 7 | 2 | | 2 | 4 | 4 | 9 | 3 | 4 | 4 | | 5 | 3 | | 3 | 2 |
| Fuzzy C-Means – *k* | | | 5 | | 8 | 7 | 2 | | 2 | 4 | 4 | 9 | 3 | 4 | 4 | | 5 | 3 | | 3 | 2 |
| DBSCAN - *eps* | | | 450 | | 300 | 150 | 12 | | 0.003 | 0.12 | 0.25 | 0.362 | 0.06 | 0.04 | 0.08789 | | 180 | 2.3 | | 0.5 | 3.5 |
| DBSCAN – *min_samples* | | | 1 | | 1 | 1 | 5 | | 11 | 5 | 5 | 1 | 5 | 3 | 2 | | 40 | 11 | | 11 | 2 |
| HDBSCAN – *min_clust_size* | | | 2 | | 3 | 2 | 3 | | 24 | 2 | 10 | 1 | 3 | 3 | 2 | | 20 | 7 | | 3 | 25 |
| HDBSCAN – *min_samples* | | | 2 | | 2 | 3 | None | | None | 10 | None | None | None | 20 | 3 | | 10 | 1 | | None | 1 |
| OPTICS - *xi* | | | 0.2 | | 0.27 | 0.34 | 0.05 | | 0.3 | 0.046 | 0.05 | 0.05 | 0.005 | 0.05 | 0.05 | | 0.005 | 0.003 | | 0.01 | 0.0001 |
| OPTICS - *min_samples* | | | 2 | | 2 | 2 | 10 | | 5 | 11 | 13 | 2 | 17 | 6 | 3 | | 40 | 10 | | 15 | 17 |
| Mean Shift - *bandwidth* | | | 400 | | 380 | 200 | 13 | | 0.0093 | 0.5 | 0.829 | 0.38 | 0.15 | 0.055 | 0.095 | | 238 | 3.9 | | 0.85 | 10.5 |
| Spectral Clustering – *k* | | | 5 | | 8 | 7 | 2 | | 2 | 4 | 4 | 9 | 3 | 4 | 4 | | 5 | 3 | | 3 | 2 |
| Spectral Clustering – *random_state* | | | 0 | | 0 | 0 | 0 | | 0 | 0 | 0 | 0 | 0 | 0 | 0 | | 0 | 0 | | 0 | 0 |
| Affinity Propagation – *preference* | | | -12616 | | -50000 | -10000 | -5000 | | -0.015 | -7.6219 | -0.15 | 0.08 | -0.0051743 | -0.00704157 | -0.008 | | -800000 | -148 | | -0.35 | -2000 |
| Affinity Propagation – *damping* | | | 0.95 | | 0.95 | 0.95 | 0.95 | | 0.95 | 0.96 | 0.95 | 0.95 | 0.95 | 0.96 | 0.95 | | 0.95 | 0.95 | | 0.95 | 0.95 |
| Gaussian Mixture Model – *k* | | | 5 | | 8 | 7 | 2 | | 2 | 4 | 4 | 9 | 3 | 4 | 4 | | 5 | 3 | | 3 | 2 |

All other parameters were the default within the cited implementations.
